# Pimavanserin Use in Lewy Body Dementia: A Case Report Demonstrating the Medication’s Efficacy

**DOI:** 10.7759/cureus.46356

**Published:** 2023-10-02

**Authors:** Michelle Baker, Wenxin Song, Adam Fusick

**Affiliations:** 1 Mental Health, University of South Florida, Tampa, USA; 2 Mental Health, University of South Florida Morsani College of Medicine, Tampa, USA; 3 Mental Health and Behavioral Services, James A Haley Veteran Affairs, Tampa, USA

**Keywords:** geriatric psychiatry, scale for the assessment for positive symptoms, parkinson disease psychosis, lewy body dementia, pimavanserin

## Abstract

Pimavanserin is an antipsychotic that is approved for use in Parkinson’s disease psychosis. Working as a serotonin 2A inverse agonist, pimavanserin allows patients to improve their psychotic symptoms without worsening the motor symptoms of Parkinson's. This mechanism is mediated via serotonin receptors and may allow for pimavanserin to be considered for use in other disease processes that present with psychosis. Here, the authors describe the case of a 75-year-old man with Lewy Body Dementia (LBD) who was started on pimavanserin. The response of the patient to the medication was measured over a six-week time course using the Scales for the Assessment of Positive Symptoms of Schizophrenia (SAPS). Overall, pimavanserin was shown to be effective in this patient with LBD. The authors also provide a review of the sparse literature attesting to other off-label uses for this unique antipsychotic.

## Introduction

Lewy body dementia (LBD) and Parkinson's disease dementia (PDD) are a group of neurodegenerative diseases that result from the accumulation of aggregated α-synuclein protein in Lewy bodies (LB) and Lewy neurites [1-3]. In addition to sharing a common neuropathological hallmark lesion (LB), they also share similar clinical features, including cognitive impairment, recurrent visual hallucinations, extrapyramidal motor features, and a history of rapid eye movement sleep behavior disorder. The major clinical distinction between LBD and PDD is based on the temporal relationship between the development of dementia relative to parkinsonism, with dementia arising in the setting of well-established Parkinson’s disease after at least one year of motor symptoms denoting PDD, while the development of cognitive impairment preceding or occurring concurrently within one year of the onset of parkinsonism denotes LBD [2,3].

Psychotic symptoms in patients with dementia are commonly seen with the progression of the disease course. Dementia-related psychosis, which includes delusions and hallucinations, contributes to institutionalization, cognitive decline, and caregiver burden [4]. While there are several off-label pharmacologic options for the treatment of psychosis, there is currently no FDA-approved medication for the treatment of psychotic symptoms in LBD [5]. Pimavanserin is an antipsychotic medication that has an FDA indication for the treatment of hallucinations and delusions associated with Parkinson’s disease. Despite this, pimavanserin is not indicated for the treatment of patients with dementia-related psychosis unrelated to Parkinson’s disease associated with hallucinations and delusions [6]. As with all antipsychotics, pimavanserin carries a black box warning of increased mortality in elderly patients (65 years of age or older) with dementia-related psychosis [7].

Pimavanserin’s mechanism of action differs from other antipsychotics on the market as it acts as an inverse agonist at the 5HT2A receptor [5]. In contrast to antagonists, inverse agonists not only block the receptor but also decrease the intrinsic activity of the receptor. In addition to the above, pimavanserin lacks affinity at dopaminergic, histaminergic, adrenergic, and muscarinic receptors, making it an attractive option for the treatment of hallucinations and delusions in LBD as it should not worsen motor symptoms or cause sedation and/or hypotension [5,8].

As neuroleptic sensitivity and the presence of hallucinations and delusions are hallmark clinical features of LBD, the authors aimed to determine the efficacy of pimavanserin over the first six weeks of initiation in a patient with LBD.

## Case presentation

A 75-year-old male veteran presented to the James A. Haley Veterans Hospital with his wife due to concerns about a lack of self-care and requesting assistance with skilled nursing facility placement. The patient was admitted to an inpatient medical service, and psychiatry was consulted for assistance with the patient’s worsening psychotic symptoms. The patient’s medical history at this time was significant for Crohn’s disease, atrial fibrillation, benign prostatic hyperplasia, hyperlipidemia, hypertension, obstructive sleep apnea, and recently diagnosed Lewy body dementia. He had no previous psychiatric history prior to admission. The diagnosis of LBD was confirmed prior to admission via dopamine transporter (DAT) scan by an outside hospital provider, and after receiving this diagnosis of LBD, galantamine 8 mg was started and tolerated well by the patient. Prior to admission, the patient was trialed on carbidopa-levodopa for worsening motor symptoms and quetiapine for worsening psychotic symptoms. Both of these were not tolerated by the patient due to worsening psychotic symptoms and increased urinary frequency, respectively.

On initial evaluation, both the patient's wife, who served as the patient's primary caregiver, and the patient himself confirmed numerous psychotic symptoms, which included visual and olfactory hallucinations and bizarre and persecutory delusions. Visual hallucinations were described as bugs and humans. Olfactory hallucinations included smelling the wife’s perfume, which she reportedly had not worn in months. Auditory hallucinations were also reported, but the patient could not elaborate on what they were saying. In addition to the above, the patient was having bizarre and persecutory delusions, which included believing he needed to pack for a trip and believing that his wife was cheating on him, respectively. The patient and wife both noted that the patient’s psychotic symptoms were causing significant distress, resulting in poor sleep. Several days into admission, the patient also developed tactile hallucinations and persecutory delusions, believing that bugs were crawling on his skin and that the nursing staff was intentionally ignoring his needs, respectively.

Psychiatry initially recommended the initiation of trazodone 50 mg, which was increased to 100 mg. After an episode of verbal agitation, trazodone was again increased to 150 mg with some improvement. Despite improvements in sleep, the patient and the patient’s wife continued to report significant distress secondary to psychotic symptoms. Pimavanserin was then initiated at 34 mg daily on day 5 of hospitalization. The efficacy of pimavanserin was measured using the Scales for the Assessment of Positive Symptoms of Schizophrenia (SAPS). The authors scored the SAPS based on the answers provided by the patient and the patient's wife. The SAPS was completed pre-initiation and at one-, two-, four-, five-, and six-weeks post-initiation of pimavanserin. The patient was deemed medically stable and discharged to a skilled nursing facility on day 12 of hospitalization, at which point the patient had completed one week of pimavanserin. He was admitted to the skilled nursing facility for 13 days prior to being discharged home.

The SAPS was chosen as the measurement tool for the efficacy of pimavanserin as it was the original screening measure used by the HARMONY trial, a phase 3 relapse-prevention study of pimavanserin in patients with dementia-related psychosis [9]. The scales were administered in person during the patient’s hospitalization and via telephone and video calls after discharge. Of note, a SAPS was not completed on week 3 post-initiation of pimavanserin at the patient's wife’s request as the appointment would fall on a holiday and the patient was in the process of transitioning back home. SAPS scores are reported in the tables below. Table 1 depicts total and global SAPS scores by category, while Table 2 focuses exclusively on a breakdown of hallucination types and their respective SAPS scores. Lastly, Table 3 shows the remaining pertinent SAPS categories and scores.

**Table 1:** Total and global SAPS scores SAPS: Scales for the Assessment of Positive Symptoms of Schizophrenia

	Pre-initiation		Week 1		Week 2		Week 4		Week 5		Week 6	
	Total	Global	Total	Global	Total	Global	Total	Global	Total	Global	Total	Global
Hallucinations	14	4	13	4	10.5	3.5	3	1	0	0	0	0
Delusions	7	4	5	2	2	2	1	1	0	0	0	0
Bizarre behavior	11	3	6	2	5	2	3	1	3	1	1	0
Positive formal thought disorder	21	3	14	2	9	2	8	2	6	2	6	2
Total	53	14	48	10	26.5	9.5	15	5	9	3	7	2

**Table 2:** Hallucinations SAPS breakdown SAPS: Scales for the Assessment of Positive Symptoms of Schizophrenia

Hallucinations SAPS breakdown	Pre-initiation	Week 1	Week 2	Week 4	Week 5	Week 6
Auditory	4	4	3	1	0	0
Voices commenting	0	0	0	0	0	0
Voices conversing	0	0	0	0	0	0
Somatic/tactile	1	0	0	0	0	0
Olfactory	0	0	0	0	0	0
Visual	5	5	4	1	0	0
Global rating	4	4	3.5	1	0	0
Total	14	13	10.5	3	0	0

**Table 3:** Delusion, bizarre behavior, and positive formal thought disorder SAP breakdown Of note, some subcategories were excluded due to pre-initiation SAP score as well as all subsequent weeks being recorded as 0. Thus, any subcategory not reported in the table above was 0.

		Pre-initiation	Week 1	Week 2	Week 4	Week 5	Week 6
Delusion breakdown	Persecutory	3	3	0	0	0	0
	Global rating	4	2	2	1	0	0
	Total	7	5	2	1	0	0
Bizarre behavior breakdown	Aggressive and agitated behavior	3	3	2	1	0	0
	Repetitive or stereotyped behavior	5	1	1	1	1	1
	Global rating	3	2	2	1	1	0
	Total	11	6	5	3	3	1
Positive formal thought disorder breakdown	Derailment	3	2	0	0	0	0
	Tangentiality	2	0	0	0	0	0
	Incoherence	4	4	3	3	2	2
	Illogicality	3	1	1	0	0	0
	Circumstantiality	3	0	0	0	0	0
	Distractible speech	3	5	3	3	2	2
	Global rating	3	2	2	2	2	2
	Total	21	14	9	8	6	6

The total SAPS score prior to the initiation of pimavanserin was 53, and by the sixth week, it had decreased to 6. At the end of week 1, auditory and visual hallucinations were largely unchanged, though the patient noted the disappearance of somatic or tactile hallucinations. Additionally, persecutory delusions were unchanged by the end of week 1, but the patient’s wife did note that the persecutory delusions appeared to affect the patient’s lifelessness, as noted by the decrease in the global rating of delusions from 4 to 2. Repetitive or stereotyped behavior rated as 5 pre-initiation was rated as 1 by the end of week 1. Improvements in positive formal thought disorder were also noted by the end of week 1, with improvements in derailment, illogicality, and global rating. Remarkably, the patient’s wife reported that distractible speech worsened by the end of week 1 as the score increased from 3 to 5. Overall, auditory hallucinations were initially noted to decrease by the end of week 2 and completely disappear by the fifth week. Visual hallucinations were also first noted to decrease by the end of week 2, and by the end of week 4, visual hallucinations were rated as 1. Ultimately, they disappeared by the end of week 5 and were rated as 0. Of note, the patient’s wife did report that while the patient was in a skilled nursing facility (between weeks 2 and 3 post-initiation of pimavanserin), he was given valproic acid, which she reported worsened the patient’s hallucinations, as hallucinations appeared to stop when the patient’s wife did not continue giving the patient valproic acid when he was discharged home from rehab at the end of week 3. Persecutory delusions were noted to disappear by the end of week 2. Additionally, week 2 saw an improvement in aggressive and agitated behavior, which was ultimately resolved by week 5. Regarding the positive formal thought disorder category, it was determined that derailment was no longer present at the end of week 2. At the end of week 6, the only symptoms remaining were in the positive formal thought disorder category, which included incoherence, distractible speech, and a global rating. All of them were each rated a 2.

## Discussion

Summarizing the data from the case presented above, SAPS scoring overall demonstrates pimavanserin efficacy in this patient. He experienced significant improvements across all symptoms between weeks 1 and 6 post-initiation of pimavanserin. The total SAPS score dramatically decreased from 53 to 6. The pimavanserin patient information guide notes that the medication may begin to reduce hallucinations as early as four weeks and that it may take up to six weeks for full effects to be seen [10]. This patient, however, started seeing benefits as early as week 1 with the disappearance of tactile or somatic hallucinations and improvements in both the bizarre behavior and positive formal thought disorder categories. Additionally, though the patient’s persecutory delusions were largely unchanged, the patient’s wife did note that these appeared to be less distressful to the patient, which was noted by the decrease in the global rating of delusions from 4 to 2. By the end of week 6, only positive symptoms of formal thought disorders and one repetitive and stereotyped behavior remained. It is important to note that the only repetitive behavior that remained was the patient taking continuous positive airway pressure (CPAP) on and off at night, and it is not clear if this was due to the repetitive and stereotyped behavior of LBD, sundowning in the evenings, or the onset of rapid eye movement (REM) sleep behavior disorder. Hallucinations and delusions were resolved by the end of week 4. By the end of the six weeks, his wife expressed “… a huge quality of life improvement.” with the resolution of both hallucinations and delusions. The patient tolerated the medication well without any adverse effects related to pimavanserin reported, which was compared to the patient's prior discontinuation of quetiapine due to the development of adverse effects. His tolerability of pimavanserin further highlights the potential benefits of this medication in patients with LBD, as neuroleptic sensitivity is a hallmark of this population.

One limitation of this study was that the SAPS scoring after the patient was discharged from the hospital was primarily dependent on our patient’s wife, a potential source of bias. Without her providing details of the patient’s behavior both in the skilled nursing facility and at home, however, complete scales would not have been able to be obtained at each follow-up encounter. Additionally, at the patient’s wife’s request, no scoring was completed for week 3, which could have provided crucial information to determine how pimavanserin efficacy may be affected by a change in living environment, as this time directly coincided with him moving from a skilled nursing facility back to his home.

Though there are some published randomized controlled studies demonstrating the efficacy of pimavanserin in the treatment of Alzheimer’s disease psychosis [11,12], to the best of the author's knowledge, only one other case report has been published demonstrating the potential benefits of pimavanserin sole use in LBD by Abadir et al. [13]. This case adds to the growing body of literature demonstrating a favorable response to pimavanserin in LBD without the adverse effects experienced with other antipsychotics. Interestingly, both our patient and the one presented by Abadir et al. showed worsening of symptoms when valproic acid was added for behavioral management [13]. What is unique about this case report, however, is the utilization of SAPS scoring, which provides invaluable information regarding specific psychotic symptoms and both how and when they responded to pimavanserin treatment. This information can aid clinicians in determining if pimavanserin may be appropriate for their patients and managing expectations of the medication's efficacy. For example, the patient presented in this case saw little improvement in incoherence and distractible speech over the six weeks but saw significant initial improvement in repetitive behavior, whereas hallucinations did not see significant improvement until week 4.

The few other studies that show pimavanserin use in this population also support the author's findings. One single-center retrospective cohort study published by Horn et al. compared quetiapine and pimavanserin for the treatment of LBD-related psychosis and found a lower early discontinuation rate in the pimavanserin cohort. It was concluded that the earlier discontinuation rate in the pimavanserin cohort suggested that pimavanserin was more likely to be effective at promptly managing psychosis compared to quetiapine, without the findings of higher mortality in the pimavanserin cohort compared with the quetiapine cohort [4]. Another study by Muller et al. looked at three patients with LBD who were placed on pimavanserin and trazodone. In line with what is presented in this case report, the combination of pimavanserin and trazodone was effective in reducing both psychotic and frontal symptoms after 6 weeks of treatment [14].

Presently, the understanding of pimavanserin use outside of Parkinson's disease is limited but growing. Reports of utilizing pimavanserin's unique mechanism of action to augment atypical antipsychotics for the treatment of psychosis as well as monotherapy for Charles Bonnet syndrome highlight the versatility this medication may have in off-label use [15-17]. Further investigation needs to be completed regarding the off-label safety of this medication, but studies of pimavanserin in its intended population have shown a decreased rate of adverse events and mortality for patients using pimavanserin compared to multiple antipsychotics [18].

## Conclusions

Overall, pimavanserin is not currently approved to treat hallucinations and delusions in LBD, but our case report demonstrates efficacy in this population. More research needs to be completed so prescribers can fully utilize this novel antipsychotic working as an inverse agonist on serotonin receptors, potentially across a wide range of different psychiatric pathologies. Despite the limited research, however, given the sparse pharmacological options available to treat LBD and based on our case report along with the literature available, pimavanserin should be considered by providers as a viable option in the treatment of hallucinations and delusions in LBD.
